# Supplementary material for: Content-rich biological network constructed by mining PubMed abstracts
Source: BMC Bioinformatics. 2004 Oct 8;5:147. doi: 10.1186/1471-2105-5-147 (PMC528731; doi:10.1186/1471-2105-5-147)
Supplement: Additional File 2 — The original results of the above study (non-essential files are deleted to keep the file size under the limit set by BMC bioinformatics). [file 1471-2105-5-147-S2.bz2 › chilibotAdditionalFile2/dip05/182ID7957082E622/html/IL5_CSF2RB.html]

 


 **IL5** and **CSF2RB** 
  
Found 2 abstracts in PubMed, retrieved 2.  
 

 What does Google say? 
 PDF only 
| .edu only 

---

**Interactive relationship** (e.g. stimulation, inhibition, etc)

**Non-interactive relationship** (e.g. studied together, co-existance, homology, etc.)

- Population distribution and pathogenetic significance for bronchial asthma BA of the eight polymorphic variants of six interleukin IL and interleukin receptor genes, C 589T, G C 3 UTR IL4, C 703T  **IL5** , T113M IL9, Q551R, 150V IL4RA, and G1972A IL5RB  [ **CSF2RB** ] , was examined.  Ref: 12575459 Genetika, 2002
